# Cardiovascular disease in hereditary haemophilia: The challenges of longevity

**DOI:** 10.1111/bjh.18085

**Published:** 2022-02-21

**Authors:** Susan Shapiro, Gary Benson, Gillian Evans, Catherine Harrison, Sarah Mangles, Mike Makris

**Affiliations:** ^1^ Oxford University Hospitals NHS Foundation Trust Oxford NIHR Biomedical Research Centre Oxford UK; ^2^ Radcliffe Department of Medicine Oxford University Oxford UK; ^3^ Belfast City Hospital Belfast UK; ^4^ Kent Haemophilia and Thrombosis Centre East Kent Hospitals University Foundation NHS Trust Canterbury UK; ^5^ Sheffield Haemophilia and Thrombosis Centre Royal Hallamshire Hospital, Sheffield Teaching Hospitals NHS Foundation Trust Sheffield UK; ^6^ Haemophilia, Haemostasis and Thrombosis Centre, Hampshire Hospitals NHS Foundation Trust Basingstoke UK; ^7^ Department of Infection, Immunity and Cardiovascular Disease University of Sheffield Sheffield UK

**Keywords:** ageing, atherosclerosis, cardiovascular, comorbidity, haemophilia, ischaemic heart disease

## Abstract

The development of effective and safe treatments has significantly increased the life expectancy of persons with haemophilia (PWH). This has been accompanied by an increase in the comorbidities of ageing including cardiovascular disease, which poses particular challenges due to the opposing risks of bleeding from haemophilia and antithrombotic treatments versus thrombosis. Although mortality secondary to coronary artery disease in PWH is less than in the general population, the rate of atherosclerosis appears similar. The prevalence of atrial fibrillation in PWH and risk of secondary thromboembolic stroke are not well established. PWH can be safely supported through acute coronary interventions but data on the safety and efficacy of long‐term antithrombotics are scarce. Increased awareness and research on cardiovascular disease in PWH will be crucial to improve primary prevention, acute management, secondary prevention and to best support ageing PWH.

## INTRODUCTION

Haemophilia is an X‐linked recessive disorder resulting in reduced or absent factor (F) VIII or FIX activity. Haemophilia is classified according to baseline FVIII/IX levels as severe (<1%), moderate (1%–5%), and mild (>5%). For persons with haemophilia (PWH) the bleeding phenotype usually corresponds to the baseline factor level, with persons with severe haemophilia (PWSH) at risk of spontaneous bleeding into joints and muscles, whereas those with moderate haemophilia tend to bleed after mild trauma, and those with mild haemophilia after more major trauma or invasive procedures.^1^ Over the last 50 years, the development and availability of clotting factor concentrates, and home treatment has allowed the rapid administration of the missing factor to stop bleeding once it occurs. Additionally, the introduction of safe concentrates in the 1990s allowing home prophylaxis, has significantly improved quality of life and life‐expectancy for PWSH. We now have PWH reaching older age, which presents new challenges with respect to managing comorbidities.^1^, ^2^ Cardiovascular disease (CVD) is the leading cause of death globally in the general population,^3^ and as PWH age they are also at risk of developing CVD. This review addresses specific issues of CVD in haemophilia.

### LIFE EXPECTANCY AND MORTALITY FROM CARDIOVASCULAR DISEASE

The twentieth century saw a marked increase in both the number of people diagnosed with haemophilia and in the survival of PWH. In Sweden, the life expectancy of PWSH increased from 11 years during 1831–1920 to 57 years during 1961–1980.^4^ In persons with moderate haemophilia the equivalent increase was from 28 to 72 years, whilst that of the general population increased from 62 to 76 years.^4^ A UK study of 6018 human immunodeficiency virus (HIV)‐negative PWH during 1977–1999 reported median life expectancy in PWSH of 63 years, whilst for moderate and mild haemophilia it was 75 years.^5^

Historically the main cause of death in PWH has been haemorrhage or complications of HIV and hepatitis C, contracted from treatment with contaminated factor concentrates.^1^ As PWH survive to older age, the causes of death have altered. A recent publication analysed the mortality in PWH from 17 papers published between 2010 and 2020.^6^ The proportion of deaths from CVD ranged from 4.8% in Italy to 25.5% in Brazil,^6^, ^7^, ^8^ with studies reporting that the proportion of deaths from CVD has increased over recent years.^7^, ^8^ Although several studies reported reduced mortality from CVD in PWH compared to the general population, events were categorised differently between datasets; that is, stroke may include both ischaemic stroke and haemorrhagic stroke, but haemorrhagic stroke is much more common in PWH.^6^ Looking specifically at mortality associated with coronary artery disease (CAD), the UK reported a rate in PWH of 62% that of the general population.^5^ Similarly, in a Dutch cohort the cause of death from CAD was reduced at 0.3 (95% confidence interval [CI] 01–0.9) even though the overall standardised mortality ratio (SMR) for PWH was 1.4 (95% CI 1.2–1.7).^9^ A systematic review of 15 studies, which included 19 242 PWH, also demonstrated a trend for reduced mortality due to arterial thrombosis compared with healthy controls (SMR 0.51, 95% CI 0.24–1.09) and this was significant when the analysis was restricted to studies with ≥10 years follow‐up (SMR 0.59, 95% CI 0.48–0.72).^10^

### HYPERTENSION

Although mortality secondary to CAD is less in PWH than in the general population, several studies in Europe and the United States (USA) have reported that PWH have a significantly higher prevalence of hypertension compared to the age‐matched male population. Analysis of 701 Dutch and UK PWH who were aged >30 years found that 49% of PWH had hypertension compared to 40% of the general population.^11^ In the USA, a study of 458 PWH aged >18 years found a similar increased prevalence of hypertension in PWH compared to the general population (overall 49% vs. 32%), which was present across all age groups.^12^ A subsequent analysis of 1431 PWH in Sweden reported an odds ratio (OR) of 2.1 for hypertension in PWH.^13^ However, a recent study of 711 PWH in Japan using centralised health records found that prevalence of hypertension was similar to males in the general population.^14^ It is noteworthy that there are methodological differences and challenges across all these studies, such as the definition of hypertension and how data were collected (retrospectively from local or centralised health records, or prospectively). Overall, the evidence is suggestive of an increased prevalence of hypertension in PWH, at least in Europe and the USA. Several of the studies reported a trend toward increased prevalence of hypertension in PWSH compared to those with mild disease,^11^, ^12^, ^13^ but this observation is not consistent across all studies.^15^ Age and body mass index were independently associated with hypertension^11^, ^12^, ^13^, ^15^ but neither HIV nor hepatitis C were.^11^, ^13^

The mechanism for the increased risk of hypertension in PWH is unknown. It has been hypothesised that PWH may suffer microbleeds into the kidneys, resulting in renovascular hypertension, fibrosis and subsequent systemic hypertension. However, neither a clear association nor causality between haematuria, renal dysfunction and haemophilia has been observed.^15^, ^16^

Few studies have published data on control of hypertension in PWH and it is of concern that the USA study reported that of the people treated for hypertension, only 27% of PWH were controlled compared to 48% in the general population.^12^

### OBESITY AND OTHER TRADITIONAL CARDIOVASCULAR RISK FACTORS

The prevalence of overweight individuals is increasing, and the World Health Organisation estimates that obesity has tripled since 1975. Meta‐analysis has shown that PWH in Europe and North America have a similar prevalence of obesity/overweight to the general population at 31%.^17^ In the general population, obesity is associated with increased risk of hypertension, type 2 diabetes, CAD and stroke. In addition, in PWH it is associated with reduced range of movement in joints and increased chronic pain,^17^, ^18^, ^19^ further reducing physical activity. The UK/Dutch study of 709 PWH found that the prevalence of diabetes and smoking were similar to the general age‐matched male population.^20^

### CARDIOVASCULAR RISK SCORES

Modelling has shown that population‐based CVD primary prevention (smoking cessation, blood pressure and cholesterol control in apparently healthy people) achieved a fourfold larger reduction in deaths in the UK compared to secondary prevention.^21^ This argues for a proactive approach to primary prevention, and cardiology guidelines recommend using a risk score to calculate cardiovascular risk over time and engaging patients in discussing modifiable risk factors.^22^ Several cardiovascular risk calculators are well‐established for the general population and there is no ‘single’ best calculator to use. Clinicians should use a locally approved a calculator, suitable for the region and ethnicity.^22^

It is not known how reliable cardiovascular risk scores are in predicting cardiovascular risk in PWH, and a couple of studies have used them to compare cardiovascular risk profiles in PWH to the general population. A Dutch/UK cohort used the QRISK(R)2 and SCORE algorithms.^20^ The predicted 10‐year QRISK(R)2 risk was significantly higher in PWH than in the general population (8.9% vs. 6.7%), indicating more unfavourable cardiovascular risk profiles; and the increased risk became apparent after the age of 40 years.^20^ In contrast, a study of 100 PWH in Europe compared to 200 aged‐matched controls found no significant difference in the 10‐year cardiovascular mortality risk >10% between PWH and controls using SCORE.^23^

### CORONARY ARTERY DISEASE

Coronary artery disease is generally subdivided into stable angina and acute coronary syndromes (ACS). Stable angina is chest pain that most often occurs with activity or stress and is associated with narrowing of the coronary vessels. ACS is a term used to describe a range of conditions associated with a sudden reduction of blood flow to the heart, that is, thrombotic occlusion following atheromatous plaque rupture. ACS comprise ST elevation myocardial infarction (STEMI), non‐ST elevation MI (NSTEMI) and unstable angina.

#### Incidence of atherosclerosis/sub‐clinical CAD disease

Although mortality secondary to CAD is less in PWH than the general population, two studies suggest that PWH develop atherosclerosis at a similar rate to the normal population. The first study measured carotid and femoral intima‐media thickness (IMT) and brachial flow‐mediated dilatation (FMD) as markers of atherosclerosis and endothelial dysfunction respectively in 51 obese PWH, and 42 obese and 50 matched non‐obese male patients.^24^ Carotid IMT was increased in obese as compared with non‐obese subjects, but no difference was found in mean carotid and femoral IMT between obese PWH and obese control individuals. In all, 35% of the obese PWH and 29% of the obese controls had an atherosclerotic plaque, irrespective of the severity of haemophilia. Brachial FMD was comparable between obese PWH and obese controls. The second study evaluated the presence and extent of atherosclerosis by coronary artery calcification score (CACS) derived from computed tomography and carotid IMT in 69 PWH.^25^ This again showed that CACS and carotid IMT were similar to controls and that the extent of atherosclerosis was related to traditional cardiovascular risk factors.^25^

#### Incidence of overt CAD

The exact prevalence of overt CAD in PWH compared to the general population is unclear. In 2015, a scoping review on the prevalence of CAD in PWH revealed 30 papers and 14 original articles, of which four indicated a protective effect of haemophilia toward CAD.^26^ The conflicting results in the original papers were considered to be potentially due to studies spanning over 30 years in publication dates, and differences in the demographics, haemophilia severity and prevalence of CAD risk factors; additionally many were small retrospective studies that can lead to ascertainment bias.^26^ In a recent 5‐year prospective multicentre study of 579 PWH, the rate of cardiovascular events was 1.7% compared to the expected rate of 4.1%. The event rate was reduced for both the severe and non‐severe haemophilia groups. In the severe haemophilia group, some of the reduction in risk was lost in patients on prophylaxis (Table 1).^27^

**TABLE 1:** Rate of cardiovascular events in haemophilia over 5 years in a prospective multicentre study

	*N*	Expected CVD events, % (*n*)	Observed CVD events, % (*n*)	Relative risk (95% CI)
Total	579	4.1 (24)	1.7 (9)	0.38 (0.18–0.80)
Haemophilia severity				
Severe	304	3.9 (12)	1.3 (4)	0.33 (0.11–1.02)
Mild	201	5.0 (10)	1.0 (3)	0.20 (0.04–0.90)
CVD risk group				
High	78	15.3 (12)	6.4 (5)	0.42 (0.15–1.13)
Intermediate	100	7.0 (7)	3.0 (3)	0.43 (0.11–1.61)
Low	401	1.5 (6)	0.2 (1)	0.17 (0.02–1.38)
Prophylaxis status^a^				
On prophylaxis	182	3.8 (7)	2.2 (4)	0.57 (0.17–1.92)
On demand	122	4.1 (5)	0 (0)	0.00

Abbreviation: CVD, cardiovascular disease.

^a^ Prophylaxis status for severe patients.

Adapted from van der Valk et al.^27^.

Overall, although the exact prevalence of overt ACS is unclear, the rate of atherosclerosis appears similar in PWH compared to the general population and yet cardiovascular mortality is reduced. One explanation for this is that in PWH reduced thrombin generation at the point of plaque rupture results in reduced risk of vessel occlusion.^28^ Another possibility is that the plaques are more stable and less likely to rupture in PWH.^29^ Evidence for this comes from mouse models; although FVIII deficiency has had inconsistent effects in different mouse models of accelerated atherosclerosis (apolipoprotein E knockout mice vs. low‐density lipoprotein receptor null mice),^30^, ^31^ hypercoagulability (induced by a mutation in the thrombomodulin gene) appears to stimulate atherosclerosis with plaques being more unstable as mice age.^32^, ^33^ Thus, it has been postulated that PWH may be protected from overt ACS and cardiovascular mortality through increased plaque stability, as well as reduced thrombin generation following plaque rupture.^29^

#### Management of CAD

Management of CAD is challenging in PWH because standard management requires antiplatelet and anticoagulant therapies, invasive procedures such as percutaneous coronary intervention (PCI), deployment of bare metal stents (BMS) or drug‐eluting stents (DES) and/or coronary artery bypass grafting (CABG) may be required for definitive re‐vascularisation. It is unclear to what extent evidence‐based guidelines for the general population^34^, ^35^, ^36^ apply to PWH.

It has been advocated to aim to treat ACS in PWH similarly to the general population with haemostatic modifications when needed.^37^, ^38^ A expert consensus publication in 2013 of management of ACS in PWH, recommended that primary PCI should be preferred to thrombolysis given the increased risk of bleeding with thrombolysis, and thrombolysis should be used only where PCI cannot be accessed.^38^ Invasive treatment of ACS should be performed as soon as possible under adequate clotting factor protection.^38^ Radial rather than femoral access for angiography was recommended, and radial access is in fact now the recommended approach in the general population due to reduction in major bleeding, vascular complications and mortality.^39^ Consensus was that trough FVIII/FIX should be around 50% within 24 h of PCI and minimum trough levels should not fall below 5%–15% on dual antiplatelet therapy (DAPT) post‐PCI.^38^ Given more recent data on bleeding risks on antithrombotics,^40^ which is discussed in more detail below, we would advocate keeping FVIII/FIX >20% on DAPT. With regards to choice of stent, historical recommendations were to use BMS in preference to DES, as it was considered that DAPT was only necessary for 1 month after BMS compared to 6 months for DES.^38^, ^41^ However, this advice has now been superseded by second‐generation DES that have higher efficacy and safety in comparison with both early‐generation DES and BMS.^36^, ^42^ Furthermore, it has recently been shown that when using the latest DES, 1 month of DAPT was not inferior to 3 months DAPT in patients at high risk of bleeding when assessed at almost 1 year, and the risk of major and clinical relevant non‐major bleeding was significantly less with abbreviated therapy (6.5% vs. 9.4%).^43^ It is reasonable to use the latest DES with 1 month of DAPT in patients with haemophilia undergoing PCI.

Published reports demonstrate that haemophilia centres can support PCI in PWH successfully. A systematic review published in 2018 reported 54 PWH undergoing PCI, the majority with BMS (57%), with only two DES and five balloon dilatations.^44^ The majority of PWH (78%) received factor replacement and were given peri‐procedural anticoagulation. PCI was successful at initial re‐vascularisation in all cases and major peri‐procedural bleeding episodes occurred in three of 54 (6%) patients. After stenting, most (85%) were treated with DAPT (median duration 1 month) and bleeding during follow‐up occurred in 11 of 54 (20%) patients.^44^

For long‐term secondary prevention, the clotting factor level at which PWH would benefit from antiplatelet agents without an unacceptably high bleeding risk is unknown. A 2‐year prospective French registry reported bleeding and thrombotic events in 68 PWH on antithrombotic therapy for CAD or atrial fibrillation (AF)^40^ and compared them to 68 matched PWH without an indication for antithrombotic treatment. The majority of recruited PWH had mild haemophilia and CAD (48 mild, 10 moderate, and 10 PWSH, 50 with ACS, 17 with AF, one with both). Antiplatelet therapy was associated with a significantly increased risk of bleeding (DAPT hazard ratio [HR] 5.3, single antiplatelet therapy HR 3.8). Bleeding was significantly higher for PWH on antithrombotics with a FVIII/FIX of <20% compared to >20% and was more in severe/moderate PWH on demand treatment as opposed to those on regular prophylaxis. Additionally, this study reported that the risk of a new cardiovascular event was at least sixfold higher in PWH with known CVD compared to those without.^40^ Therefore, antithrombotic treatment is indicated in PWH with known CVD, but the balance of bleeding and thrombosis is challenging. An individual bleeding history should be considered when deciding on antithrombotic therapy and patients should be carefully counselled and regularly reviewed. The recent data would support using factor prophylaxis if baseline FVIII/FIX is <5% for as long as an antithrombotic drug is prescribed. They also highlight the significant bleeding risk in those with FVIII/FIX of 5%–20% where factor replacement should be considered.

## ATRIAL FIBRILLATION

### Prevalence

A European study of 3952 PWH found the overall prevalence of AF was 0.84% and increased with age (0.2% at ≤60 years, 3.4% at >60 years).^45^ Although this prevalence is similar to the historical prevalence in the general population (0.95% overall, 3% in people aged 65–69 years and 9% in those >80 years),^46^ the current prevalence of AF in adults is reported as 2%–4%,^3^ and a twofold increase is expected due to the increasing age of the general population and also improved detection of AF.^47^ It is therefore unclear as to whether the prevalence of AF in PWH is similar to the general population or lower. The European study found the prevalence of AF was significantly lower in PWSH compared to non‐severe haemophilia (0.4% [seven of 1760] vs. 1.1% [26/2192]); however, rather than being a true difference in risk this may simply relate to the older age on average of non‐severe PWH in the study (27% vs. 11% aged >60 years).

#### Antithrombotics to reduce stroke risk

In the general population, international guidelines advise anticoagulation for stroke prevention in AF if the CHADS2VASC score in men is ≥1, unless the bleeding risk is particularly high.^47^, ^48^ Anticoagulation is associated with a two‐thirds reduction in stroke risk and a reduction in death by a quarter.^47^ Either vitamin‐K antagonists, such as warfarin, or direct oral anticoagulants (DOACs) may be suitable anticoagulants following patient counselling, although many advocate the use of DOACs first line due to both ease of use and 50% reduction in the risk of intracranial haemorrhage.^47^ Antiplatelet drugs are no longer recommended to reduce the risk of stroke in AF.^47^ It is recommended to use bleeding risk tool scores such as HAS‐BLED (hypertension, abnormal renal and/or liver function, stroke, bleeding, labile international normalised ratio [INR], elderly, drugs or alcohol)^49^ or ORBIT (older age, reduced haemoglobin, bleeding history, insufficient kidney function, treatment and antiplatelet)^50^ to identify and address potentially modifiable risk factors such as uncontrolled hypertension that might otherwise increase the risk of bleeding.^47^

Data on stroke risk secondary to AF in PWH have not yet been published. As PWH have reduced haemostatic potential, it is likely that the risk of stroke will be less than for the general population for an equivalent CHADS2VASC score.^51^ Coupled with the fact that PWH have a higher bleeding risk when started on antithrombotic therapy,^40^ PWH are likely to have a different threshold for starting antithrombotics than the general population. In an effort to understand where this threshold might be, de Konging et al. ^52^ explored the thrombin generation potential of 133 PWH A (15 severe, 118 non‐severe) compared to people on vitamin‐K antagonists and healthy controls. Severe haemophilia was associated with a similar thrombin potential as therapeutic warfarin (INR ≥2); however, non‐severe haemophilia was associated with higher mean thrombin potential, and a third of patients had thrombin potential above the upper cut‐off of patients on therapeutic anticoagulation (30% of people with FVIII 1%–19%, and 48% of those with FVIII 20%–50%). This suggests that antithrombotic therapy for AF should be considered in non‐severe PWH, particularly those with FVIII/FIX of >20%. Although thrombin potential is not a true global test of haemostasis and it is not known how this translates to in vivo thrombotic and bleeding risk, a factor threshold of 20% is consistent with the observations of the French antithrombotic registry discussed previously.^40^ Although the number of people with AF in this French registry was limited (*n* = 18), the number of patients who reported bleeding episodes was significantly higher in patients with a HAS‐BLED score of ≥3 than in those with a HAS‐BLED score of <3 (five of eight patients vs. none of 10 respectively), whereas the median clotting factor level of patients with HAS‐BLED scores of ≥3 and <3 was similar (16.5% vs. 19.5%) as well as the proportion of patients on prophylaxis.

Previous consensus recommendations based on expert opinion have differed, but mainly suggest considering anticoagulation when FVIII/FIX is >20%–30%.^53^, ^54^ Based on more recent data it may be reasonable to reduce this level to 20% in people with HAS‐BLED scores of <3 but the bleeding and thrombosis risks are finely balanced. DOACs are recommended over vitamin‐K antagonists due to reduced risk of major bleeding and intracranial haemorrhage.^54^ Patients with high CHADS2VASC scores and factor levels of <20% are a particularly challenging group and antiplatelet monotherapy has previously been recommended.^54^ Given that antiplatelet monotherapy has not been shown to be effective in AF in the general population and yet is associated with significant increased bleeding risk in PWH, we would not advocate using this for AF in PWH. Non‐anticoagulant options and the efficacy of low‐dose DOACs should be investigated in PWH with AF.

#### Non‐anticoagulant options

Several procedures are possible that avoid the need for long‐term anticoagulation and these are attractive in PWH.

Cardioversion in association with a short acting general anaesthetic can be used to revert to sinus rhythm. This is usually more successful in newly presenting AF. When AF has been present for >48 h, anticoagulation is likely to be required for 4 weeks before and after the procedure.

Catheter ablation, using a femoral vein approach, can be used to interrupt the abnormal electrical activity causing the AF and can be effective at maintaining sinus rhythm in patients with AF.^47^ In the general population its main indication is for symptom control; however, for patients considered at high risk of bleeding (HAS‐BLED ≥3) it has been shown to be as effective as anticoagulation in preventing long‐term thromboembolic complications but with less risk of major bleeding.^55^ Van der Valk et al.^56^ described a single‐centre experience of eight procedures in five PWH and one with von Willebrand disease. All patients obtained long‐term sinus rhythm (in two patients after a second procedure), although one relapsed at 42 months. Groin bleeds were experienced after two of eight (25%) of the procedures, which is higher than the rate observed in persons without a bleeding disorder (0.9%).^56^

Left atrial appendage occlusion using one of several approved devices may also be considered in PWH. The Watchman device was non‐inferior to vitamin‐K antagonists for stroke prevention in randomised controlled trials in the general population with a possibility of lower bleeding rates on long‐term follow‐up.^57^ However, the implantation procedure can have serious complications and antithrombotics post‐procedure have not been evaluated in randomised controlled trials but usually consist of an antiplatelet agent.^47^ Experience in haemophilia is limited and a recent systematic review of the world literature identified only nine patients.^58^ There was significant variability in the device used, severity of haemophilia as well as the type of antithrombotic therapy used initially after the procedure. Although the procedure itself was uncomplicated in all cases, two PWSH treated with clopidogrel had bleeding complications.^58^

## CARDIAC SURGERY

With immediate and plentiful availability of clotting factor concentrates, a multidisciplinary haemophilia team and an onsite specialist coagulation laboratory, any surgery including cardiac surgery, can be carried out safely in PWH.^59^ Despite this, less invasive procedures of equal efficacy are preferred such as the use of PCI instead of CABG, transcatheter correction of septal defects and valve implantations and use of off cardiopulmonary bypass (CBP) procedures.^60^, ^61^, ^62^

As for all surgery in PWH, certain principles must be adhered to, and these are described in Table 2. Although many haemophilia centres use a continuous factor infusion regimen, it is equally safe to use bolus administration of concentrate twice daily with an extra dose, if required, at the end of the surgery.^63^ The administration frequency of concentrate postoperatively will depend on whether a standard or extended half‐life product is used.

**TABLE 2:** Key issues in the management of cardiac surgery in patients with haemophilia without inhibitors

Preoperative	Measure baseline clotting screen. Measure FVIII/IX levels. Test for inhibitor to FVIII/IX. Multidisciplinary team meeting to agree surgery plan.
On day of the operation	Use minimally invasive surgery, if possible. Use a bioprosthetic valve for valve replacement if possible. Surgery with either bolus injections or continuous infusion of factor concentrate is acceptable. Infuse FVIII or IX and measure pre and post FVIII/IX levels. Provided levels are >0.80 iu/ml surgery can proceed. If using cardiopulmonary bypass: Standard heparinisation and reversal with protamine Monitor heparin use with the ACT or APTT For patients on emicizumab Measure FVIII levels with a bovine chromogenic assay Monitor heparin with anti‐Xa or non‐ACT/APTT assay.
Postoperative	Measure FVIII/IX levels at least once daily. Aim for FVIII/IX to be >0.80 iu/ml for first 72 h. Aim for FVIII/IX to be >0.50 iu/ml for the following 5–7 days. Antiplatelet and anticoagulant drugs can be used but moderate and severe haemophilia patients should be on factor prophylaxis.

Abbreviations: ACT, activated clotting time; APTT, activated partial thromboplastin time; F, factor.

Here we will specifically consider the procedures of coronary artery bypass grafting (CABG), heart valve replacement and heart transplantation.

### CABG

The number of these procedures carried out has reduced significantly in recent years. Off‐CPB procedures are possible and should be used in preference, if available.^62^ The outcome of CABG in PWH has been reported to be good in the papers published to date. Rossi et al.^64^ summarised 25 papers describing a total of 28 patients and although the results were positive the authors speculate that there could be publication bias of the cases with good outcome being published. A major issue with CABG in haemophilia is the monitoring of the anticoagulation on CPB. Provided FVIII/IX has been normalised, anticoagulation should be with unfractionated heparin in the usual manner with activated clotting test (ACT) monitoring. Normally there is no need to measure FVIII/IX whilst the patient is on CPB, but if this is required it can be done with chromogenic assays.^60^ Patients on emicizumab pose a particular challenge and are discussed below.

### Valve replacement

For PWH who require a valve replacement, two particular issues that should be considered are whether the surgery can be carried out using transcatheter aortic valve implantation (TAVI) and the need for long‐term anticoagulation if a mechanical prosthesis is used.^60^, ^61^ Patients with mechanical valves require life‐long anticoagulation with vitamin‐K antagonists and therefore a bioprosthetic valve is the preferred choice for the majority of PWH,^65^, ^66^ although mechanical valves have been employed.^67^

### Heart transplantation

Very few of these procedures have been carried out in PWH but the principles are those of any cardiac surgery using CPB.^68^ One potential issue is that many of the heart transplant centres do not have a haemophilia centre onsite but with appropriate collaboration, safe surgery is possible.

There are certain situations when cardiac surgery in haemophilia is more challenging, and this includes surgery in patients with inhibitors to FVIII/IX and patients on emicizumab.

### Cardiac surgery on patients with inhibitors

Surgery on patients with inhibitors is challenging at any time, and cardiac surgery on CPB is particularly so. In patients with a low level of inhibitor, higher doses of FVIII/IX may be used but frequent factor level measurements will be required, and continuous infusion is often preferred. If FVIII/IX cannot be used, then bypassing agents such as recombinant FVIIa or FEIBA (Factor Eight Inhibitor Bypassing Activity) should be employed.^60^ As the activity of by‐passing agents cannot be easily measured, replacement is formulaic. For some patients, especially those with acquired haemophilia A, recombinant porcine FVIII concentrate can be used if the patient does not have inhibitors to porcine FVIII.

### Patients on emicizumab

The use of emicizumab is rapidly increasing in resource rich countries and is used in patients with haemophilia A with or without inhibitors. Patients on emicizumab have a very short activated partial thromboplastin time (APTT), which means that assays based on this cannot be used to measure FVIII levels. FVIII levels during replacement therapy with FVIII concentrate need to be measured with a bovine chromogenic assay. During CPB, the APTT and ACT cannot be used to monitor the heparinisation of the circuit, so conventional anti‐Xa assays or the point of care Hepcon protamine titration device should be used.^69^

## AREAS FOR FUTURE RESEARCH

In the UK, the James Lind Alliance Priority Setting Partnership for bleeding disorders reported in 2019.^70^ It was the first robust research priority setting partnership in bleeding disorders that included the opinions of patients, carers, and healthcare professionals. The second of the top 10 priorities was: ‘How can we balance the risk and benefit of antithrombotic treatment for cardiovascular disease in patients with bleeding disorders?’, which highlights both the current uncertainty in managing these patients, but also the recognition of the importance of research in this area by both patients and healthcare professionals.

In addition to the issues already raised in this review, haemophilia treatment is rapidly advancing, and this may affect the rate of thrombosis following plaque rupture. The target FVIII/IX trough levels with prophylaxis are now higher than in the past and this may increase the thrombotic risk of patients with severe and moderate haemophilia. Furthermore, the development of novel agents such as emicizumab, and others in ongoing clinical trials that re‐balance haemostasis such as Fitusiran (small interfering RNA to anti‐thrombin) and Concizumab (anti‐tissue factor pathway inhibitor), may impact on the prothrombotic potential and CVD events. Thrombotic microangiopathy and thromboembolism occurred in emicizumab‐treated patients receiving repeated infusions of activated prothrombin complex concentrates.^71^ In the absence of additional activated prothrombin complex concentrate, there is uncertainty as to the risk of CVD events with emicizumab, because in the clinical trials there were relatively few people aged >65 years, and known CVD was an exclusion criterion.^72^, ^73^, ^74^ A recent publication of emicizumab in 17 PWH aged >50 years, including nine patients with multiple CVD risk factors (four of whom had known CAD and were on concomitant antiplatelets) found that it was well tolerated and no thrombotic events were reported.^75^ Real‐word data over time will be required to establish the risk of CVD events with emicizumab in the ageing population. Although thrombotic events have been reported in some of the trials of re‐balancing agents, the extent of the increased thrombotic risk of any future licensed product, and any effect of underlying cardiovascular risk factors on this, currently remains unknown. Key areas of future research, including the impact of novel agents, are highlighted in Table 3.

**TABLE 3:** Areas of future research for haemophilia and cardiovascular disease

Mortality	Standardisation of mortality data reporting. Regular (e.g. 5 yearly) reviews of causes of death in PWH: are there changes due to the ageing population, increasing CVD risk factors, changing prophylaxis and use of novel agents or gene therapy?
CVD risk factors	Aetiology of increased prevalence of hypertension in PWH. Control of risk factors such as hypertension and dyslipidaemia in PWH compared to the general population.
CAD	Improving understanding of pathogenesis of ACS and plaque stability in haemophilia including clinically silent atherosclerosis: novel non‐invasive imaging techniques, *in vitro* and animal models. Increased accuracy of risks factors, prevalence, outcome of CVD and optimal therapy likely through large prospective international registries/trials e.g. (a) Incidence of clinically apparent CAD and how improving standard CVD risk factors affect this. Is this changing over time due in increasing cardiovascular risk factors, changing prophylaxis and novel agents? (b) Incidence of bleeding and recurrent thrombosis with long‐term use of antithrombotic therapy.
AF	Better data on prevalence of AF, risk of stroke secondary to AF in PWH, and long‐term optimal therapy likely through large prospective international registries/trials. Does this change over time due to changing prophylaxis regimens and novel agents?

Abbreviations: ACS, acute coronary syndromes; AF, atrial fibrillation; CAD, coronary artery disease; CVD, cardiovascular disease; PWH, persons with haemophilia.

## CONCLUSION

It has been acknowledged for many years that the haemophilia population is ageing and that the safe and effective management for CVD will become an increasing issue. Recent publications of prospective registry data are very helpful and show the significant increased bleeding risk associated with antithrombotic therapy, as well as recurrent CVD events in PWH. Given the challenges of treating known CVD with antithrombotics, haemophilia teams should promote awareness of cardiovascular risk, and primary prevention programmes with healthy life‐style advice and optimal management of known risk factors such as hypertension, diabetes and high cholesterol. They should also enable national and international prospective registry data to improve our understanding of prevalence and best management, as well as monitoring how changing haemophilia prophylaxis, including with novel therapies, will affect CVD.

## CONFLICT OF INTEREST

Catherine Harrison has received conference support, educational speaker and/or consultancy fees from Roche, Sobi, Pfizer, Novo Nordisk, Takeda, CSL Behring, Sanofi. Sarah Mangles has received conference support, educational speaker fees and/or advisory board fees from SOBI, Roche, NovoNordisk, Takeda, CSL Behring and Octapharma. Susan Shapiro has received conference support, educational speaker fees, and/or advisory board fees from Pfizer, Bayer, Shire, Takeda, Roche and CSL Behring. Gary Benson, Gillian Evans and Mike Makris have no potential conflicts of interest in relation to this manuscript.

## AUTHOR CONTRIBUTIONS

All authors conceived and designed the review; each undertook a literature review and drafted a section of the review. Susan Shapiro drafted the manuscript and critically revised it with Mike Makris. All authors approved the final version.
